# Latitudinal consistency of biomass size spectra - benthic resilience despite environmental, taxonomic and functional trait variability

**DOI:** 10.1038/s41598-020-60889-4

**Published:** 2020-03-05

**Authors:** Mikołaj Mazurkiewicz, Barbara Górska, Paul E. Renaud, Maria Włodarska-Kowalczuk

**Affiliations:** 1 Institute of Oceanology Polish Academy of Sciences, 81-712 Sopot, Poland; 2 Akvaplan-niva, Fram Centre for Climate and the Environment, 9296 Tromsø, Norway; 3 University Centre in Svalbard, 9171 Longyearbyen, Norway

**Keywords:** Climate-change ecology, Community ecology, Marine biology

## Abstract

Global warming is expected to cause reductions in organism body size, a fundamental biological unit important in determining biological processes. Possible effects of increasing temperature on biomass size spectra in coastal benthic communities were investigated. We hypothesized higher proportions of smaller size classes in warmer conditions. Soft bottom infauna samples were collected in six Norwegian and Svalbard fjords, spanning wide latitudinal (60–81°N) and bottom water temperature gradients (from −2 to 8 °C). Investigated fjords differed in terms of environmental settings (e.g., pigments or organic carbon in sediments). The slopes of normalised biomass size spectra (NBSS) did not differ among the fjords, while the benthic biomass and NBSS intercepts varied and were related to chlorophyll *a* and δ^13^C in sediments. The size spectra based on both abundance and biomass remained consistent, regardless of the strong variability in macrofauna taxonomic and functional trait composition. Variable relationships between temperature and body size were noted for particular taxa. Our results indicate that while benthic biomass depends on the nutritional quality of organic matter, its partitioning among size classes is consistent and independent of environmental and biological variability. The observed size structure remains a persistent feature of studied communities and may be resilient to major climatic changes.

## Introduction

Body size is a fundamental biological characteristic that determines basic life-processes of organisms, including metabolic rate, generation time or locomotion speed^1^. In multispecies assemblages, size structure can define species interactions, including position in food webs, and pathways and magnitudes of carbon flow through the system components^2,3^. In aquatic studies, size spectra, i.e., abundance or biomass distribution among size classes^4,5^, are frequently assessed for communities, since such structural organization may have stronger implications for functionality than commonly reported taxonomic composition, diversity or abundance^6^. The knowledge about the size distribution of organisms in the ecosystem may be useful not only as its descriptor but also as a basis for size-based ecosystem modelling to assess eco-evolutionary processes or possible consequences of environmental transformations due to human-induced changes^7^. Yool *et al*.^8^ incorporated knowledge of benthic biomass partitioning among size classes in modelling and in predictions concerning possible changes in the shelf and deep-sea ecosystems and forecasted a substantial decline of deep-sea benthic communities biomass (−32%) in future scenario of high greenhouse gas emissions.

Size spectra may be powerful indicators of ecosystem functioning (e.g., productivity) and environmental changes (e.g., anthropogenic disturbances)^9,10^. Despite their widespread and growing use in pelagic studies, size spectra remain rarely explored in marine benthos, mostly due to methodological difficulties and time consuming laboratory sample processing (measurements of hundreds of specimens). Early reports go back to 1980s when Schwinghamer^11^ and Warwick and Gee^12^ described the characteristic modal size distribution of benthic community components. Recent benthic size-spectrum studies investigated the impact of various environmental drivers operating at local scales^13,14^.

Parameters (intercept and slope) of linear regressions of normalized (i.e., divided by the width of the size class^15^) biomass to size class are commonly used as size spectrum descriptors. When a community function in an ecosystem in a steady state (lack of disturbance, the flux of energy is from smaller to larger organisms) the slope coefficient should be close to −1^16^. Pelagic systems of the Sargasso Sea or the central gyre in the North Pacific Ocean are examples of such steady state marine systems^5,17^. Deviations from the steady state may be visible in changes of the linear regression slope coefficient of normalized biomass size spectrum (NBSS) or in variation of residuals along regression lines^16^. In particular, the NBSS slope coefficient was shown to decrease with increasing trophic state of the system i.e., from oligotrophic to eutrophic along with growing variation of linear regression residuals^18^. In subsidized systems like deep-sea detritus-based benthic communities or estuaries with high allochthonous inputs from land, a slower biomass decrease with body size (NBSS slope coefficient >−1) may be observed^19,20^. In such systems size structure does not reflect trophic relationships because organisms share the same base of resources. It is different from most pelagic systems, where higher trophic levels (larger organisms) obtain energy by eating smaller prey. For example deep-sea megafauna does not evince the typical Eltonian reduction in biomass with increasing, as many species feed by scavenging, not by consuming smaller organisms^21^. Regarding benthic communities, NBSS parameters were shown to be sensitive to various stressors^22^ and related to environmental gradients^6,13^.

Ecosystem functioning is more strongly influenced by changes in the size structure than changes in taxonomic community diversity^23^. Changes in the size structure of multispecies assemblages may have substantial consequences regarding interactions among organisms. For example, one driver of ecosystem functioning change may be a mismatch in relative sizes of prey and consumer organisms. A decline in the average size of primary producers not accompanied by corresponding changes in consumers may potentially result in the shrinkage of food resources for heterotrophs and then, a reduction in consumer populations and increased susceptibility to diseases or higher mortality^24^. Strong proportional increase of contributions of smaller classes to total biomass may lead to an increase of total secondary productivity^14^. On the other hand, Norkko *et al*.^3^ showed that the presence of large organisms in soft-bottom benthic communities determines the intensity of bioturbation and bioirrigation, factors that supply deeper parts of sediment with organic matter and oxygen. The importance of size in bioturbation effects was underlined by Canfield and Farquhar^25^, who estimated that the evolution of bioturbators resulted in a several-fold increase of sulphate concentration in the ocean. Thus, the role of size in determining ecosystem functioning, and the possibility that climate change may potentially shift community size structure suggests this is an important (but largely neglected) characteristic of ecosystems.

Declining body size has been proposed as one of the three universal ecological consequences of climate warming, alongside changes in species distribution and phenology^26^. Theory on the links between body size and climate/latitude was based on observations of endothermic organisms, where Bergmann’s rule states that animals from cooler climates tend to be larger than those from warmer climates^27^. Further studies on ectotherms led to the development of a temperature-size rule^28,29^, which states that there is a negative relationship between rearing temperature and organism final body size. Several studies support predictions of climate warming effects on organism size in aquatic systems. For instance, maximum body size in marine fish assemblages is expected to shrink up to 24%, due to changes in distribution, abundance and physiology^30^. A meta-analysis of long-term surveys, experimental data, and published results concerning the effect of climate change on the body size of aquatic bacteria, phytoplankton, zooplankton and fish showed that global warming may impact community size structure in various ways; e.g., increasing the proportion of small species or young age-classes, or causing a decline in size-at-age of distinct taxa^31^. For example, in eastern Fram Strait mesozooplankton, the proportion of the small, boreal hyperiid amphipod *Themisto compressa* increased while that of the large, Arctic *T. libellula* decreased with increasing water temperatures between 2000 and 2012^32^. Furthermore, mesocosm studies in freshwater pelagic ecosystems have shown that warming of 4 °C may cause significant changes in size structure. For example, increasing the prevalence of small phytoplankton organisms and altering the energy flow in the ecosystem^33^, or in benthic communities, decreasing the total biomass and the proportion of large size classes^23^. Certainly, other scenarios are also possible, e.g. disappearance of large organisms (with no change in other organism’s biomass) that may narrow the size class range but does not alter the NBSS slope. Also, if average size of all components of the community is impacted in the same way^31^, only the NBSS intercept changes. Heterogeneous changes across different components of the community may result in more irregular (less linear) relationships^34^.

Our aim was to investigate possible consequences of climate warming for Arctic benthic community size structure. We used a “space-for-time” approach, i.e., treatment of the lower latitude/warm localities as proxies of future/after warming situation in the Arctic. We compared the biomass size spectra in coastal (fjordic) soft bottom habitats in six localities across latitudinal/thermal gradients, spanning from 60 to 81°N latitude and 8 to −2 °C bottom water temperature. We expected that warmer thermal conditions may influence size structure of benthic fauna through size reduction of organisms within dominant species or changing species composition promoting smaller organisms. We hypothesized that: (1) size structure of benthic fauna will change along the thermal/latitudinal gradient by increasing contributions of smaller size classes or decreasing proportions of large organisms that will be indicated by steepening of the slopes of NBSS in warmer localities and (2) the NBSS intercept and community biomass will be related to food availability, as indicated by fresh organic matter content in sediments (indicated by pigment content, carbon content and δ^13^C). At particular taxa levels, we expected the latitudinal clines in size would be in accordance with the temperature-size rule theory (smaller bodies in warmer fjords).

## Results

### Environmental variability

**Figure 1:**
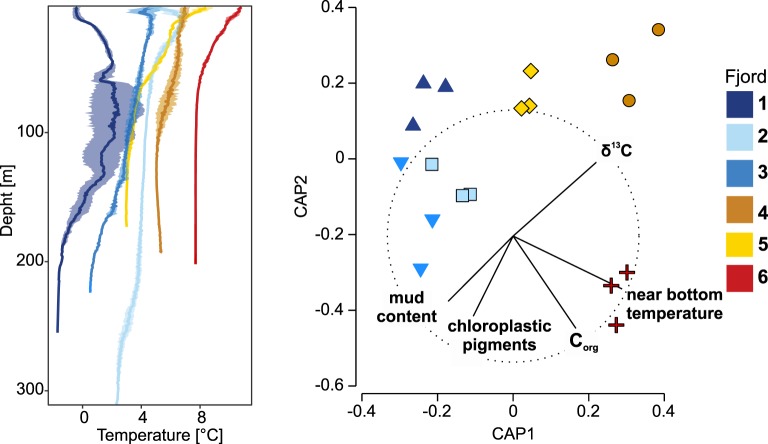
Temperature profiles (mean and SD for three stations) in fjords (left), canonical analysis of principal components (CAP) ordination based on sediment characteristics and near bottom temperature (right). Vectors on the CAP plot indicate variables with Pearson correlation to CAP axes >0.7 (C_org_ – organic carbon in sediments).

Thermal conditions in the water column differed among the studied fjords. The strongest vertical gradients were noted in fjords 3, 5 and 6. Maximum temperature values were observed in surface waters in fjords 3, 4, 5, and 6, while the warmest temperatures in fjords 1 and 2 were in intermediate water layers (Fig. 1). The average near-bottom temperature in fjords varied along latitudinal gradient (from 7.7 °C in the southernmost fjord to −1.6 °C in the northernmost fjord), except for fjord 5 (lower than in fjord 4) and fjord 3 (lower than in fjord 2). Near-bottom salinity varied between 33.4 and 35.3 (with no latitudinal pattern) among the fjords (Supplementary Table S1). Average C_org_ in surface sediments was in the range of 1.7–2.0% in most fjords, except for fjord 6 (3.8%, Supplementary Table S1). δ^13^C was lower in fjord 3 (−23.6) than in other fjords (−22.3 to −21.2). Mean values of Chl *a* were highest in fjord 3 (9.9 µg g^−1^), intermediate in fjords 2 and 5 (5.2 and 6.8 µg g^−1^, respectively), and much lower in other locations (1.7–2.0 µg g^−1^). Mean values of chloroplastic pigments were also the highest in fjord 3 (55.1 µg g^−1^), relatively high in fjord 2 (50.2 µg g^−1^), lower in fjords 5 and 6 (28.6–31.3 µg g^−1^) and very low in fjords 4 and 1 (15.1–18.9 µg g^−1^). Mud content ranged from 70 to 90% in all localities except for fjord 4 (41%). Two main gradients of environmental variability can be observed from the ordination based on the canonical analysis of principal coordinates (with the first two canonical axes explaining 74% of the variance): the difference between fjord 6 and other fjords (defined by C_org_ and near-bottom water temperature, Fig. 1) and a gradient from fjord 3 to 4 (defined by chloroplastic pigments, δ^13^C and mud content). Spitsbergen fjords (1, 2, 3) form one cluster, defined by high chloroplastic pigments concentrations and low temperatures. Fjords 4 and 5 can be described as moderately warm fjords, with high δ^13^C and low chloroplastic pigments content. Fjord 6 is the warmest and the richest in C_org_, with moderate chloroplastic pigments content and δ^13^C.

#### Benthic standing stocks and size spectra

Total benthic dry mass (DM) differed among fjords (PERMANOVA p < 0.05, Supplementary Table S2). DM was higher in fjord 3 (42.5 g DM m^−2^) than in fjords 4 and 6 (5.3 and 9.3 g DM m^−2^, respectively), and higher in fjord 2 (15.4 g DM m^−2^) than in fjord 4 (*post hoc* pairwise tests, p < 0.05). Macrofauna constituted the majority of benthic DM (91 to 99%). δ^13^C, Chl *a* and mud content were related to variability in total benthic DM (DistLM marginal test, p < 0.05), while Chl *a* and δ^13^C were selected by a forward selection procedure as the two factors explaining most of the variability (41%).

**Figure 2:**
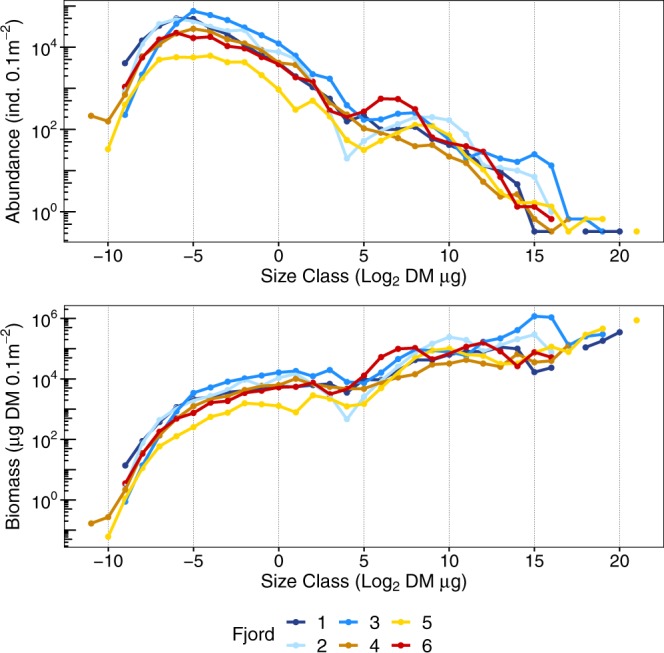
Abundance and biomass size spectra in fjords.

Organisms in the samples spanned a range of size classes from −11 to 21 (meiofauna −11 to 5, macrofaunal nematodes −6 to 6, macrofauna 0 to 21; Supplementary Fig. S1). The largest size classes (>17) were represented only in fjords 1, 3 and 5. The general shapes of both abundance and biomass spectra were similar among fjords: two distinct modes were evident – one for meiofauna and one for macrofauna (Fig. 2). The low point between modes (size classes 2–5) was very prominent in fjords 2 and 5 and less evident in fjords 1 and 4. A macrofaunal peak in abundance size spectra was observed between size classes 7 and 10 in all fjords except fjord 6, where it shifted towards lower size classes (6–7, caused by the very high abundance of polychaete *Pseudopolydora paucibranchiata*). The size class with the highest biomass occurred on the end of each spectrum, except fjords 2 and 3, where they were was observed in the penultimate size classes (size class 15).

**Figure 3:**
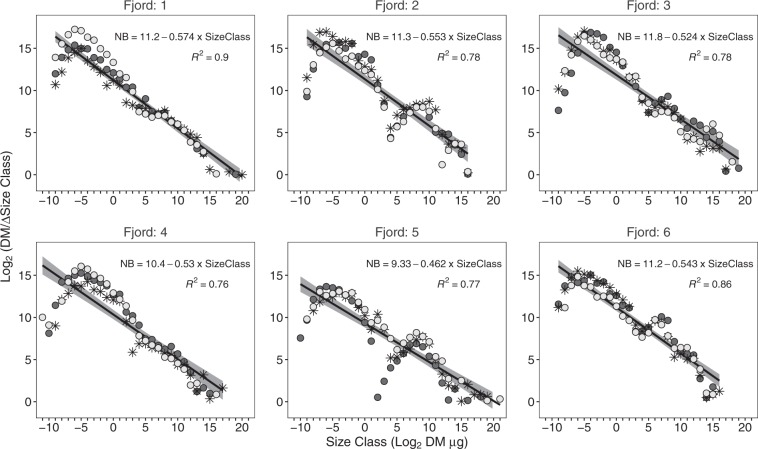
Normalized biomass size spectra (NBSS) in fjords. Symbols represent three replicate stations. Solid lines represent NBSS regression lines with standard errors.

Consistent regressions between normalized biomass and size classes for every fjord were documented by the NBSS plots (p < 0.05, Fig. 3). Slopes of NBSS for each fjord ranged from −0.46 ± 0.03SE in fjord 5 to −0.57 ± 0.02SE in 1, with no differences among localities (ANCOVA F_5, 457_ = 1.80, p = 0.11). However, intercepts differed among fjords (ANCOVA F_5, 457_ = 12.25, p < 0.001). To study differences among intercepts, a multiple linear regression with fixed slope (−0.53 ± 0.01SE) was calculated. The highest intercept was in fjord 3 (11.77 ± 0.24SE), which was higher than in fjords 4 (10.35 ± 0.27SE) and 5 (9.45 ± 0.33SE, Tukey-adjusted pairwise *post hoc* comparisons p < 0.05). The intercept in fjord 5 was lower than in all other fjords except for fjord 4.

NBSS intercepts did not correlate with total benthic DM (ρ = 0.22, p = 0.39) or mean DM (ρ = 0.15, p = 0.54). However, when only the size class range common to every fjord (from – 10 to 16) was considered, both total DM and mean DM were correlated with NBSS intercepts (ρ = 0.69, p < 0.01 and ρ = 0.52, p = 0.03, respectively).

### Taxonomic and functional trait composition in macrofauna

**Figure 4:**
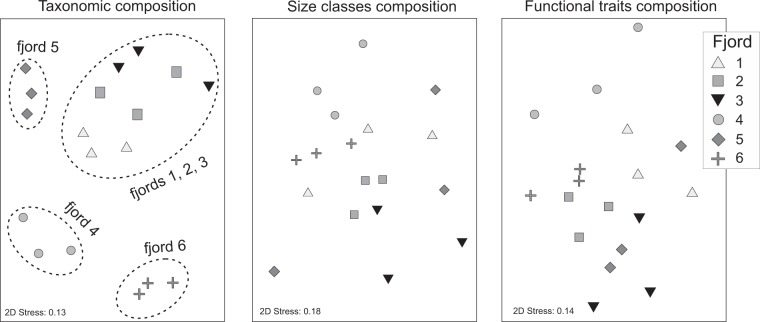
Non-metric multidimensional scaling (nMDS) of Bray-Curtis similarities of square root transformed data of macrobenthic biomass partitioned into taxa, size classes, and functional traits.

Patterns of similarity among stations differed depending on whether ordinations were based on environmental data (Fig. 1) or macrofauna biomass data partitioned among taxa, size classes and functional groups (Fig. 4). The lack of congruence among respective similarity matrices was also confirmed by RELATE analysis^35^. Moderate matching was found only between environmental variability (canonical analysis of principal coordinates, Fig. 1) and macrofauna species composition variability, and between functional group variability and size class variability (rho = 0.6, p = 0.001, in both cases). Other combinations of similarity matrices exhibited much lower (poor) correlations (rho = 0.3–0.4, p < 0.05). The nMDS plot, based on taxonomic composition, showed four station groups: (1) fjord (6, 2) fjord (4, 3) Svalbard fjords (1, 2, 3), (4) fjord 5 (Fig. 4). No clear clusters of stations could be distinguished on ordinations based on size classes or feeding groups, indicating that variability among stations was similar or even higher than variability among fjords.

**Figure 5:**
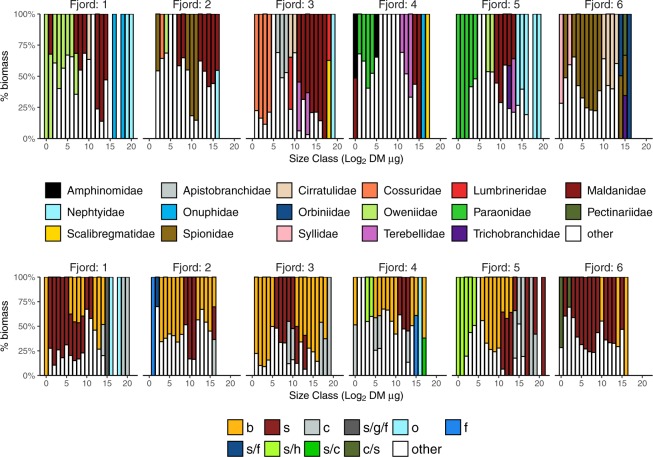
Percentage contributions of polychaete families and feeding groups to biomass in macrofaunal size classes in fjords. All groups that constituted <30% in each size class were classified as ‘other’. Feeding groups: b – subsurface deposit feeders, c – carnivores, s – surface deposit feeders, o – omnivores, f – suspension feeders, h – herbivores, g - grazers.

Polychaeta dominated all macrofaunal size classes, except for the smallest classes (1–5) in fjord 1 (dominated by Mollusca). Taxonomic composition of polychaetes varied considerably among the fjords (Fig. 5). In fjord 1, four families dominated: Oweniidae (small size classes), Maldanidae (middle size classes), Onuphidae and Nephtyidae (the largest size classes). In fjord 2, the composition of most size classes was diverse, but larger size classes consisted mostly of Maldanidae and Spionidae. In fjord 3, small size classes were dominated by Cossuridae, while large size classes were dominated by Maldanidae. In fjord 4 the polychaete composition was the most diverse, and no evident dominance was observed. In fjord 5, small size classes were dominated by Paraonidae, middle classes were dominated by Maldanidae, and the largest by Nephtyidae. In fjord 6, most size classes were dominated by either Cirratulidae or Spionidae.

Three feeding types prevailed in macrofauna (subsurface deposit feeders, surface deposit feeders and carnivores) in all locations, with varying proportions among the fjords and size classes (Fig. 5). Additionally, in fjord 5, a considerable proportion of surface deposit feeder/herbivores was noted in small size classes. No consistent pattern in the composition of size classes, in terms of feeding types, was noted among fjords. For example, carnivores dominated large size classes in fjords 1 and 5, while in 3 they were spread from moderate size classes to the largest, and in 4 they contributed to almost the entire range of size classes (Fig. 5).

### Body size vs temperature in macrofaunal taxa

Only four macrofaunal species were present in all studied fjords: *Heteromastus filiformis, Leitoscoloplos mammosus, Levinsenia gracilis*, and *Pholoe assimilis*. For *L. mammosus* and *L. gracilis*, a relationship of decreasing body mass with increasing temperature was noted (GLM, p < 0.05, Supplementary Table S3), while for *H. filiformis* the trend was opposite (p < 0.05); no trend was noted for *P. assimilis*. At the genus level, we noted 9 taxa present in every fjord; positive relationships between DM and temperature were found for three (*Heteromastus, Lumbrineris* and *Yoldiella*), negative relationships were found for two (*Chaetozone* and *Nephtys)*, and no relationship was found for four (*Chone, Diplocirrus, Microclymene* and *Pholoe)*. Fourteen families with occurrences in every fjord were noted. A positive relationship between DM and temperature was documented for four (Capitellidae, Cirratulidae, Lumbrineridae and Yoldiidae), while negative for six (Ampharetidae, Maldanidae, Paraonidae, Sabellidae, Spionidae and Terebellidae), and no relationship was found for four (Flabelligeridae, Nephtyidae, Orbiniidae and Pholoidae).

## Discussion

The general shape and the slope of benthic biomass size spectra did not vary across the wide geographical and thermal range sampled in this study. Therefore, we reject the hypothesis (1) of an increase in the proportion of smaller size classes and steepening of the slope of normalized spectra towards warmer waters. The invariance in the partitioning of biomass among size classes was observed for both abundance and biomass size spectra and despite differences in species or functional (feeding) group composition and environmental conditions in studied localities. Thus it seems that main characteristics of the size structure (shape and slope of size spectra) remain inherent features of stable and undisturbed marine soft-sediment communities. This suggests these systems are resilient to natural environmental variability, including thermal regime shifts observed at present spatial scales and predicted to occur in the near future^36^.

Our estimates of NBSS slopes agree with values reported in other benthic studies (in most cases between −0.5 and −1.3)^6,13,37,38^. Significant differences in benthic size spectra may appear in response to various anthropogenic and natural disturbances. For example, hypoxic conditions can negatively affect the slope of benthic NBSS (slope ~−0.8 in oxygen minimum zone vs. slope ~ −0.5 outside oxygen minimum zone)^22^. In another case, lower values (steepening) of the slope due to reduced presence of large organisms along bathymetric gradients across continental margins were attributed to decreasing food availability and food supply predictability^38^. Similarly, in a gradient related to river influence (decreasing food and increasing mineral matter input), NBSS slopes for deltaic macrofauna decreased from −0.5 in river mouth to −1.0 in the plume area^13^. Increasing proportions of smaller size classes in benthic size spectra have also been reported, due to glacial disturbances (high sedimentation rate of mineral material, sediment instability) in Arctic fjords^14^ and for a fish community in a sewage-enriched river ecosystem^39^.

On the other hand, in the absence of a severe stressor, size spectra appear insensitive to natural variability. Duplisea and Drgas^40^ showed similar size structures in areas of different grain size composition, despite the importance of this driver of variability in taxonomic composition of benthic biota. High consistency and independence from a range of environmental conditions (days since the last storm, carbon and nitrogen content in sediments, grain size) of size spectra were also reported for benthic communities by Schwinghamer^41^. In the present study, neither the temperature nor the other variables (grain size, organic matter content and characteristics) effected the distribution of benthic biomass among the size classes. Mazurkiewicz *et al*.^42^ reported seasonal constancy in benthic size spectra in the outer basin of west Spitsbergen fjord (Kongsfjorden). Sprules^43^ analysed multiannual changes in pelagic biomass size spectra in the Laurentian Great Lakes and did not report any significant differences in size spectra (despite substantial changes in species composition), biological invasions, or alterations in water quality. He also reported a lack of differences in size spectra among lakes of different geological ages, biological, chemical and physical properties.

Our research differed from earlier findings. Górska and Włodarska-Kowalczuk^14^ suggested a shift in the macrofaunal size spectrum towards higher size classes at lower temperatures/higher latitudes. They compared benthic biomass size spectra, constructed based on their own Arctic collections, with published studies from lower latitudes^12,37,44^. However, our study, performed on materials collected over a wide geographical range and analysed in a standardized way, did not confirm such a trend. We noted that organisms in the largest size classes (>17) were present only in colder fjords (1, 3, 5). This agrees with theories of organism size clines along thermal gradients (e.g., Bergmann’s rule, temperature-size rule)^27,31^. Admittedly, in each case there were only few specimens present in the largest size classes, but the size class extent agrees with other benthic studies that used similar sized or even smaller sampling gear and reported organisms up to 2 g DM, i.e. our size class 20^13,38,40^. Whereas our sampling stations generally fall within one biogeographical province^45,46^ there is a considerable variation across this latitudinal range in dominant water masses (coastal water masses along the Norwegian mainland, modified Atlantic Water in fjord 2, Arctic Water in fjord 1 and 3), bottom water temperature (from −2 to 8 °C), and in magnitude and seasonality of primary production (lower annual primary productivity and highly seasonal production and delivery of high quality food at more northerly latitudes). Further, structural and functional groups present in the different areas along the sampled range differed^47,48^. Thus, within the sampled geographical range, we detected no evidence for substantial latitudinal shifts in size-structure, although sampling across a broader latitudinal range, spanning more biogeographical provinces may yield different results; further studies on a larger scale seem warranted.

Minor differences in size spectra among fjords can be related to local phenomena. For example, in fjord 6, a peak between size classes 6 and 7 in abundance size spectra was due to the high dominance of the polychaete *Pseudopolydora paucibranchiata*, an invasive species originating from Pacific Japan^49^. Individuals of *P. paucibranchiata* can reach very high densities and are able to exclude other tube-building polychaetes^50^.

Much of the variability in biomass and the intercepts of the NBSS model in the present study was explained by δ^13^C and Chl *a* (confirming hypothesis 2). Both parameters are regarded as indicators of organic matter composition and origin. Thus, our study supports the notion that quality rather than quantity of food determines the level of benthic productivity. As reported in numerous studies, sediment pigment concentration, which represents the ‘freshly produced’ fraction of organic matter in sediments and can be a measure of ecosystem productivity, was linked with higher biomass^51–53^. The relationship between δ^13^C and benthic standing stock is less obvious, especially in this study, where we reported higher standing stock in fjords characterized by lower δ^13^C values. Depleted δ^13^C is usually interpreted as indicating a higher contribution of terrestrial or partially decomposed organic matter^54,55^. In the studied locations δ^13^C was higher in northern Norway fjords (low precipitation and terrestrial inflows) than in southern Norway (high precipitation and dense land vegetation) and Svalbard (glacial erosion)^56^. Considerable amounts of such terrestrially derived/processed material may be an important buffer of the highly seasonal phytoplankton production for benthic systems capable of assimilating this detritus^57^.

The intercepts of the NBSS differed among fjords (from 9.45 to 11.77). According to Guiet *et al*.^58^, NBSS intercepts decrease with increasing temperature if ecosystem resources are constant. This is due to the imbalance between food assimilation of organisms and their mortality and metabolism that increase with warming. In our study, resources differed among fjords (both in terms of quantity and quality, as indicated by C_org_, sediment pigment concentrations and δ^13^C), and those differences were not related to the latitude/temperature regime. In the NBSS model, the intercept is regarded as an indicator of community biomass^18^ or ecosystem richness in food reserves^58^. However, according to Hua *et al*.^37^, NBSS intercepts cannot be accepted as a universal indicator of total biomass, as they are sensitive to the size class range. Our data indicate that the intercept can be treated rather as a proxy of the total (or average) biomass only when compared communities are consistent in terms of the size class range.

The high contribution of macrofauna to the total benthic DM was similar across the studied fjords (91–99%) and agreed with those reported from other soft-bottom systems^59^. Górska and Włodarska-Kowalczuk^14^ explored the effects of food and disturbance on benthic biomass partitioning among size groups in Arctic fjords and reported a similar range in the contribution of macrofauna (81–94%), regardless of the level of food availability. Only in the presence of strong mineral sedimentation at glacier fronts did this proportion drop strongly, to approximately 60%^14^. Thus, even at this crude resolution (meiofauna, macrofauna), our data still suggest the partitioning of biomass among groups of organisms defined by size criteria to be a consistent feature of benthic communities, provided stable sediments and undisturbed conditions.

Species composition varied among the studied locations resulting most likely both from differences in regional species pools and environmental conditions in particular fjords that act as filters, shaping the composition of taxa^48^. The regional constraints are visible e.g., in the close location of stations from three Spitsbergen fjords on the ordination. The two north Norwegian fjords are quite different in terms of species composition, despite their close geographic location, most likely due to their different environmental character: fjord 5 is heavily silled and filled with colder water, whereas fjord 4 is open and has warmer, oceanic water masses. Interestingly, even if a polychaete family (e.g., Maldanidae) was dominant/subdominant in several locations, it was abundant in different size classes in each location, indicating the independence of taxonomy and size composition.

The functional (feeding type) group composition was independent of geographical location, and more closely reflected the differences in species composition. In addition, no persistent pattern appeared in the distribution of feeding types across macrobenthic size classes. One of the properties of pelagic food webs is the relatively consistent relationship between predator and prey body sizes^43^, suggesting the size structure of pelagic ecosystems can be used to predict the efficiency of predator-prey interactions and standing stock^60^. In benthic food webs, feeding links can also be size-based, i.e., smaller organisms being consumed by larger ones^61^. However, the overall pattern is more complicated, due to the wide size range of primary consumers of organic matter. This was also observed in this study, where no tendency of increasing (with size class) contribution of predators to the biomass was noted, as for example in fjords 3 or 5, where carnivores composed mostly small and intermediate macrofauna size classes. This suggests that energy flow in benthic food webs has more complicated pathways compared to pelagic food webs, which are linked in a chain of progressively larger size classes from planktonic producers to large predators.

The concept of increasing body size with increasing latitude/decreasing temperature (Bergmann’s rule)^27^ has been supported by numerous marine and terrestrial examples. For instance Atkinson^28^ found negative effect of elevated temperature a on body size of 83.5% of 109 reviewed organisms. Similarly, in marine crustaceans Timofeev^62^ confirmed decreasing temperature as a driver of increasing body size manifested both along latitudinal and water depth gradients. Additionally, studies on Atlantic calanoid species from contrasting temperature regimes showed not only interspecific variation in body size, but also intraspecific ones, with significantly larger organisms in High Arctic compared to Norwegian fjords^63^. Regarding terrestrial organisms, ants from the British Isles exhibited increased body size with increasing latitude; however, the strength of this trend depended on the subfamily^64^. Declining body size with increasing temperature has been also investigated with regard to climate warming^24^, e.g., in the British common toad (*Bufo bufo*)^65^ or *Mesodesma mactroides* clams of the Uruguayan coast^66^. A similar pattern was also observed for marine ostracods from the genus *Poseidonamicus*, which have increased in size more than 50% during the last 40 million years, corresponding to climate cooling^67^. However, a trend towards publishing only significant trends may distort the accumulated scientific evidence^68,69^. In the present study, we explored this relationship for all taxa with a wide enough distribution and found no consistent relationship between temperature and body size at various taxonomic levels, from species to family. A similar outcome was achieved for different butterfly families from North America, Australia, Europe and Africa^70^. In the case of benthic fauna, the reverse (or lack of) responses of body size to changing latitudes/thermal conditions in particular taxa resulted in an invariance in size structure, explored at the whole community level, as opposite effects equalized each other.

## Conclusions

Size structure was a conservative feature of benthic communities of undisturbed soft bottom sediments. It was insensitive to natural variability in both environmental (temperature and food availability) and ecological (total biomass, taxonomic composition, and functional trait composition) characteristics of the benthic systems. On the species level, the effects of temperature on body size were inconclusive, and the responses of individual taxa did not translate into changes visible at the whole community level. This indicates that size structure of coastal benthic communities may be an inherent property, highly adaptable to different conditions and will not be affected by thermal regime shifts caused by climate change. This may also represent a form of resilience that will lead to the maintenance of ecological functioning, especially in terms of energy transfer in the food web and geochemical processes in sediments. Still, there may be communities prone to changes in size structure due to climate warming. Inner basins of fjords will suffer increasing mineral matter supply and sedimentation due to increasing meltwater discharge^71^. This may cause a reduction of large organisms in these areas resulting in a shift towards a dominance of meiofaunal biota in functioning and processing of organic matter^14^.

## Materials and Methods

### Sampling

**Figure 6:**
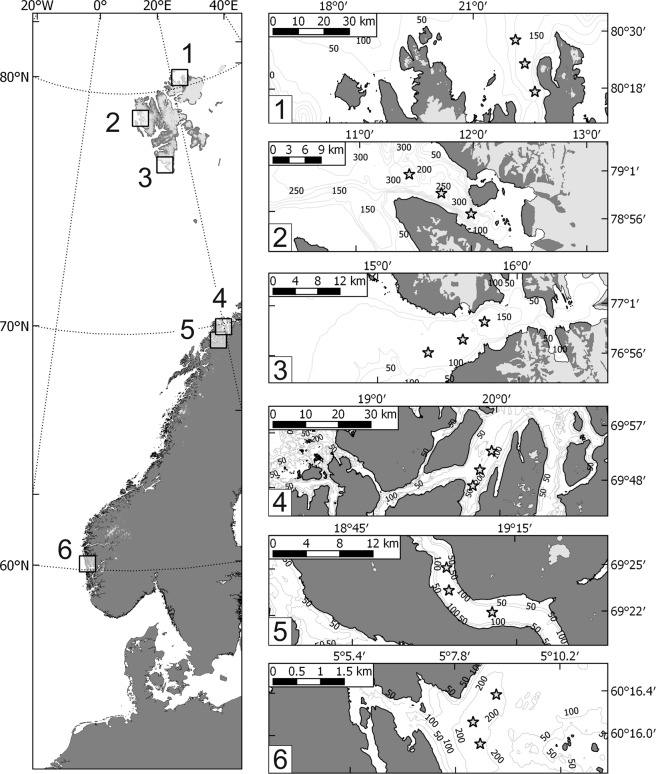
Sampling locations (squares, left panel) and sampling stations in fjords (asterisks, right panel). 1- Rijpfjorden, 2 – Kongsfjorden, 3 – Hornsund, 4 – Ullsfjorden, 5 – Balsfjorden, 6 – Raunefjorden.

Materials were collected in six fjords off Svalbard (Rijpfjorden – 1, Hornsund – 2, Kongsfjorden – 3), and continental Norway (Ullsfjorden – 4, Balsfjorden - 5 and Raunefjorden - 6, Fig. 6). They were selected to span wide latitudinal (60 to 81 °N) and thermal (bottom water temperature −2 to 8 °C) ranges (Supplementary Table S1) and to avoid variability in other environmental pressures, strong anthropogenic impacts or red king crab predation (*Paralithodes camtschaticus*), which can reduce abundance of benthic biota^72^. In each fjord, sampling was conducted at three stations, with seabed covered with fine sediments in the central part (150–350 m depth) of the outer basin (to exclude the influence of local glacial or fluvial inflows, usually located in the fjords’ heads^48^).

Materials were collected from R/V “Oceania” and R/V “Helmer Hanssen” in 2014 and 2015 (Supplementary Table S1). A set of measurements and samples collected at each of three stations included CTD profiles, sediment samples (three replicates for photosynthetic pigment concentration, one replicate for grain size, δ^13^C and particulate organic carbon (C_org_) content), one macrofauna sample and one meiofauna sample. Samples from the surface (0–1 cm and 1–2 cm) sediment layers were collected with a Niemisto gravity corer and frozen (samples for photosynthetic pigment analysis at −80 °C; other samples at −20 °C). Macrofauna was sampled with the use of a 0.1 m^2^ van Veen grab and sieved on board through 500 µm mesh; meiofauna was sampled with the use of a plastic syringe (10 cm^2^ sampling area) inserted 10 cm deep into sediment collected with a box-corer. Faunal samples were preserved in 4% formaldehyde. The three meiofauna and macrofauna samples collected at three stations were treated as replicate samples for these localities. Replicated sampling within stations (usually performed in diversity assessments) was not conducted due to: (1) time constraints and the duration of laboratory analyses, and 2) in an earlier study, no difference in size spectra between replicates collected at the same station was evident in statistical treatment of data^14^.

### Laboratory analysis

Chlorophyll *a* (Chl *a*) and phaeopigment (together referred to as chloroplastic pigments) concentrations in sediment samples were measured fluorometrically, with the use of a Perkin Elmer LS55 Fluorescence Spectrometer. Grain size composition was determined with a Malvern Mastersizer 2000 particle size analyser, recalculated using GradiStat 4.0. software. δ^13^C and C_org_ content analyses were performed via continuous flow - elemental analysis - isotope ratio mass spectrometry (CF-EA-IRMS), at the University of Liège, with the use of a Vario Micro Cube elemental analyser.

The meiofauna samples were centrifuged three times in a solution of colloidal silica (Ludox TM-50, density of 1.18 g cm^−3^) and stained with Rose Bengal in a 4% buffered formaldehyde solution. Next, samples were sieved, and specimens that passed through 500 µm mesh and retained on 32 µm mesh were analysed. Nematodes retained on 500 µm mesh were termed ‘macrofaunal nematodes’. Organisms were identified to the lowest possible taxonomic level. Each specimen was photographed with a Leica DFC450 digital camera, connected to a Leica M205C stereomicroscope and, except for nematodes, measured with Leica LAS Manual Measurements software. Nematodes were measured using a semi-automated method of image analysis^73^. For macrofaunal species occurring in numbers higher than 250 per sample, a subsample of 200 randomly picked specimens was measured. For meiofauna nematodes subsamples of 500 randomly selected individuals were analysed.

### Statistical analyses

Canonical analysis of principal coordinates was used to assess environmental parameters (near bottom temperature, C_org_, δ^13^C, mud content – contribution of silts and clay, Chl *a*, chloroplastic pigments; mean values per station) that best discriminated studied fjords.

The biovolumes of organisms were calculated based on measured dimensions of specimens. For non-nematode meiofauna, biovolume was calculated with the use of the Feller and Warwick formula^74^: V = L*W^2^*c, where V is the volume, L - length, W - width, and c - taxon-specific coefficient. For Nematoda, a formula for a volume of cylinder was used^73^. In the case of fragmented polychaetes, L was estimated using empirically determined relationships between widths of selected chaetigers and intact specimen length^75^. Wet mass (WM) was calculated by multiplying V by a specific gravity factor of 1.13^76^. For Crustacea and Ophiuroidea, WM was obtained from measured dimensions, using published conversion factors^77^. Dry mass (DM) for meiofauna was estimated as 0.25*WM^74^. Body mass conversion factors^78^ were used for obtaining macrofaunal DM. Organisms were grouped into size classes based on individual DM [μg] on a log_2_ scale. Each size class is two times wider than the previous one e.g., size class 4 includes DM values that are ≥2^4^ (16 µg) and <2^5^ (32 µg), and the size class 4 width is 16 µg (∆size class). Abundance and biomass of meiofaunal organisms and macrofauna nematodes in each size class was summed and standardized to the area of 0.1 m^2^. Next the data were combined by summing meiofauna, macrofauna nematodes and macrofauna data in corresponding size classes.

Differences in total DM among fjords were tested using one-way PERMANOVA based on a similarity matrix created from the Euclidean distances among samples^79^. Monte Carlo resampling was used to increase the interpretability of the test. To identify the best set of environmental variables accounting for the variation in total benthic DM, a distance-based linear model (DistLM, marginal and sequential tests with forward selection procedure of predictors and adjusted R^2^ selection criterion and 9999 permutations) was used^79^.

The patterns of similarity in community structure described by taxonomic composition, size class composition and functional (feeding type) trait composition among stations were explored for macrofauna. Species were classified into feeding types based on available literature^80–82^. DM data were square-root transformed, and Bray-Curtis similarities among stations were visualized using non-metric multidimensional scaling (nMDS). The relations among the three resemblance matrices was assessed using RELATE analysis^83^.

Normalized biomass (NB) was calculated by dividing total biomass in the n^th^ size class by the n^th^ size class width (∆size class) e.g., total biomass in size class 4 was divided by 16 µg. Next, normalised biomass size spectra (NBSS^5,15^) were constructed for each fjord using linear regression. Regression models were inspected for influential points by visual analysis of residuals and Cook’s distance assessment, in particular the lowest and highest size classes, which could arise from a limited number of samples, as recommended by Sprules & Barth^16^. Especially the underestimation of extreme size classes could influence the NBSS coefficients calculations and produce incorrect results.

To identify spatial variation in NB, we used an analysis of covariance (ANCOVA), with size class as a continuous covariate and fjord as a factor covariate. The multiple linear regression was used to assess the parameters (slope and intercept) of obtained NBSS. Pairwise *post hoc* comparisons were performed with a Tukey’s adjustment of p-values^84^.

The relationship between NBSS intercepts (calculated for each station separately), total benthic DM at each station, and NB averaged over size classes at each station, was tested using Spearman’s ρ rank correlation.

To explore the relationship between near-bottom temperature and DM of individual taxa, we applied a generalized linear model (GLM) with a gamma distribution and log-link. Tests were performed for species, genera and families that were observed in all fjords.

Canonical analysis of principal coordinates, PERMANOVA, nMDS, RELATE and DistLM analyses were performed using Primer v7 + Permanova^79,83^, the rest of the analyses used R 3.5.1.^84,85^.

## Supplementary information

Supplementary information.

Supplementary information 2.

## Data Availability

The datasets analyzed during the current study are available from the corresponding author on request.
