# Supplementary material for: Latitudinal consistency of biomass size spectra - benthic resilience despite environmental, taxonomic and functional trait variability
Source: Sci Rep. 2020 Mar 5;10:4164. doi: 10.1038/s41598-020-60889-4 (PMC7057973; doi:10.1038/s41598-020-60889-4)
Supplement: Supplementary file 1 — Supplementary information. [file 41598_2020_60889_MOESM1_ESM.pdf]

Supplementary Information

Latitudinal consistency of biomass size spectra - benthic resilience despite environmental, taxonomic and functional trait variability

Mikołaj Mazurkiewicz[1*], Barbara Górska[1], Paul E. Renaud[2, 3], Maria Włodarska-Kowalczuk[1]

1) Institute of Oceanology Polish Academy of Sciences, 81-712 Sopot, Poland
2) Akvaplan-niva; Fram Centre for Climate and the Environment; 9296 Tromsø, Norway
3) University Centre in Svalbard; 9071 Longyearbyen, Norway

Supplementary Table S1 Sampling details: fjord coordinates and date and environmental characteristics of these localities: near-bottom water temperature (NBT) and salinity (NBS), chlorophyll *a* (chl *a*), chloroplastic pigments**,** particulate organic carbon (C_{org}), carbon stable isotope composition ($\delta^{13}C$) and mud content (silts and clay) content in sediments. Mean ± SD are presented.

Fjord	Lat [°N]	Long [°E]	Date [year.month]	NBT [°C]	NBS	C_{org} [%]	$\delta^{13}C$	Chl *a* [µg g^{-1}]	Chloroplastic pigments [µg g^{-1}]	Mud content [%]
1	80.20	22.07	2014.09	-1.578 ± 0.124	34.780 ± 0.054	1.77 ± 0.22	-22.13 ± 0.36	2.00 ± 0.72	15.10 ± 3.31	70.4 ± 21.3
2	78.58	11.72	2014.07	2.300 ± 0.047	34.840 ± 0.045	1.77 ± 0.51	-22.60 ± 0.30	5.29 ± 2.22	55.08 ± 12.26	91.0 ± 6.4
3	76.97	15.60	2014.08	1.672 ± 0.839	35.345 ± 0.039	1.82 ± 0.13	-23.61 ± 0.17	9.86 ± 5.57	50.19 ± 17.50	82.5 ± 1.2
4	69.84	19.85	2014.06	5.313 ± 0.072	34.400 ± 0.052	1.71 ± 0.27	-21.16 ± 0.51	1.65 ± 0.56	18.83 ± 19.36	41.2 ± 1.6
5	69.38	19.04	2015.06	3.000 ± 0.013	33.400 ± 0.014	1.97 ± 0.36	-21.68 ± 0.12	6.81 ± 3.43	28.26 ± 15.73	80.8 ± 5.8
6	60.27	5.13	2015.06	7.700 ± 0.003	34.869 ± 0.001	3.85 ± 0.23	-22.27 ± 0.04	1.65 ± 0.43	31.28 ± 7.48	73.5 ± 10.8

Supplementary Table S2 Abundance and biomass of meiofauna, macro nematodes and macrofauna in fjords. Mean ±SD values are presented. Fjords with significantly lower or higher values (PERMANOVA *post hoc* pairwise test $p<0.05$) are listed in brackets

Fjord	Group	Abundance [indiv. 0.1m^{-2}]	Biomass [µg DM 0.1m^{-2}]	Contribution to total biomass [%]
1	Meiofauna	224 433 ± 209 435	41 384 ± 27 552	3.2
2		244 600 ± 130 739	55 021 ± 20 230 (>5)	3.6
3		302 767 ± 60 368	101 155 ± 13 025 (>5, 6)	2.4
4		137 333 ± 69 387	42 952 ± 19 588	8.1
5		37 000 ± 7 671	8 800 ± 3 202 (<HSD, KGF)	0.4
6		111 167 ± 43 940	30 423 ± 16 122 (<HSD)	3.3
1	Macro nematoda	1 033 ± 1 185	3 824 ± 2 746	0.3
2		1 767 ± 2 281	6 086 ± 6 906	0.4
3		1 367 ± 551	13 864 ± 10 645	0.3
4		767 ± 404	3 490 ± 1 293	0.7
5		650 ± 212	5 661 ± 270	0.2
6		733 ± 306	3 870 ± 1 321	0.4
1	Macrofauna	841 ± 146	1 241 330 ± 775 101	96.5
2		998 ± 355	1 481 438 ± 414 739	96.0
3		1 431 ± 730	4 139 356 ± 2 091 792	97.3
4		586 ± 149 (<3, 6)	481 456 ± 259 653	91.2
5		569 ± 236 (<6)	2 332 508 ± 164 0240	99.4
6		2 086 ± 483 (>4, 5)	898 092 ± 120 558	96.3

Supplementary Table S3 Results of generalized linear models (family=Gamma, link=log) testingrelationship between near bottom temperature and indyvidual body size (DM) for macrofauna species, genera and families that occurred in all fjords.

			Estimate	Std. error	t	p
Species	*H. fillformis*	Intercept	4.35	0.06	77.85	<0.001
		Temp	0.03	0.01	2.58	0.010
	L. mammosus	Intercept	5.73	0.05	123.96	<0.001
		Temp	-0.14	0.02	-8.54	<0.001
	L. gracilis	Intercept	4.22	0.08	49.78	<0.001
		Temp	-0.13	0.02	-6.98	<0.001
	P. assimilis	Intercept	5.08	0.19	26.16	<0.001
		Temp	0.04	0.04	0.90	0.369
Genus	*Chaetozone*	Intercept	6.59	0.07	92.24	<0.001
		Temp	-0.07	0.03	-2.96	0.003
	Chone	Intercept	4.32	0.12	37.18	<0.001
		Temp	-0.07	0.04	-1.82	0.072
	Diplocirrus	Intercept	7.60	0.19	40.99	<0.001
		Temp	0.02	0.04	0.63	0.531
	Heteromastus	Intercept	4.33	0.05	82.39	<0.001
		Temp	0.03	0.01	2.82	0.005
	Lumbrineris	Intercept	6.10	0.05	113.63	<0.001
		Temp	0.08	0.02	4.69	<0.001
	Microclymene	Intercept	6.05	0.08	71.27	<0.001
		Temp	0.05	0.03	1.38	0.170
	Nepthys	Intercept	12.99	0.49	26.45	<0.001
		Temp	-0.59	0.13	-4.47	<0.001
	Pholoe	Intercept	5.07	0.18	28.50	<0.001
		Temp	0.05	0.04	1.55	0.123
	Yoldiella	Intercept	5.39	0.06	90.01	<0.001
		Temp	0.09	0.02	3.87	<0.001
Family	Ampharetidae	Intercept	8.10	0.34	23.52	<0.001
		Temp	-0.49	0.08	-6.27	<0.001
	Capitellidae	Intercept	4.49	0.14	31.76	<0.001
		Temp	0.08	0.03	2.85	0.005
	Cirratulidae	Intercept	6.38	0.06	107.55	<0.001
		Temp	0.14	0.02	9.21	<0.001
	Flabelligeridae	Intercept	7.60	0.19	40.99	<0.001
		Temp	0.02	0.04	0.63	0.531
	Lumbrineridae	Intercept	6.13	0.07	85.63	<0.001
		Temp	0.08	0.02	3.53	0.000

	Maldanidae	Intercept	8.86	0.09	96.51	<0.001
		Temp	-0.19	0.03	-5.78	<0.001
	Nephtyidae	Intercept	11.73	0.38	30.94	<0.001
		Temp	-0.16	0.11	-1.43	0.160
	Orbiniidae	Intercept	5.57	0.11	52.15	<0.001
		Temp	0.05	0.04	1.55	0.121
	Paraonidae	Intercept	5.04	0.15	32.75	<0.001
		Temp	-0.19	0.03	-6.35	<0.001
	Pholoidae	Intercept	5.07	0.18	28.50	<0.001
		Temp	0.05	0.04	1.55	0.123
	Sabellidae	Intercept	5.33	0.26	20.15	<0.001
		Temp	-0.21	0.08	-2.51	0.013
	Spionidae	Intercept	8.03	0.20	40.70	<0.001
		Temp	-0.26	0.04	-6.92	<0.001
	Terebellidae	Intercept	8.95	0.19	47.59	<0.001
		Temp	-0.17	0.06	-2.73	0.007
	Yoldiidae	Intercept	5.54	0.06	97.47	<0.001
		Temp	0.21	0.02	10.77	<0.001

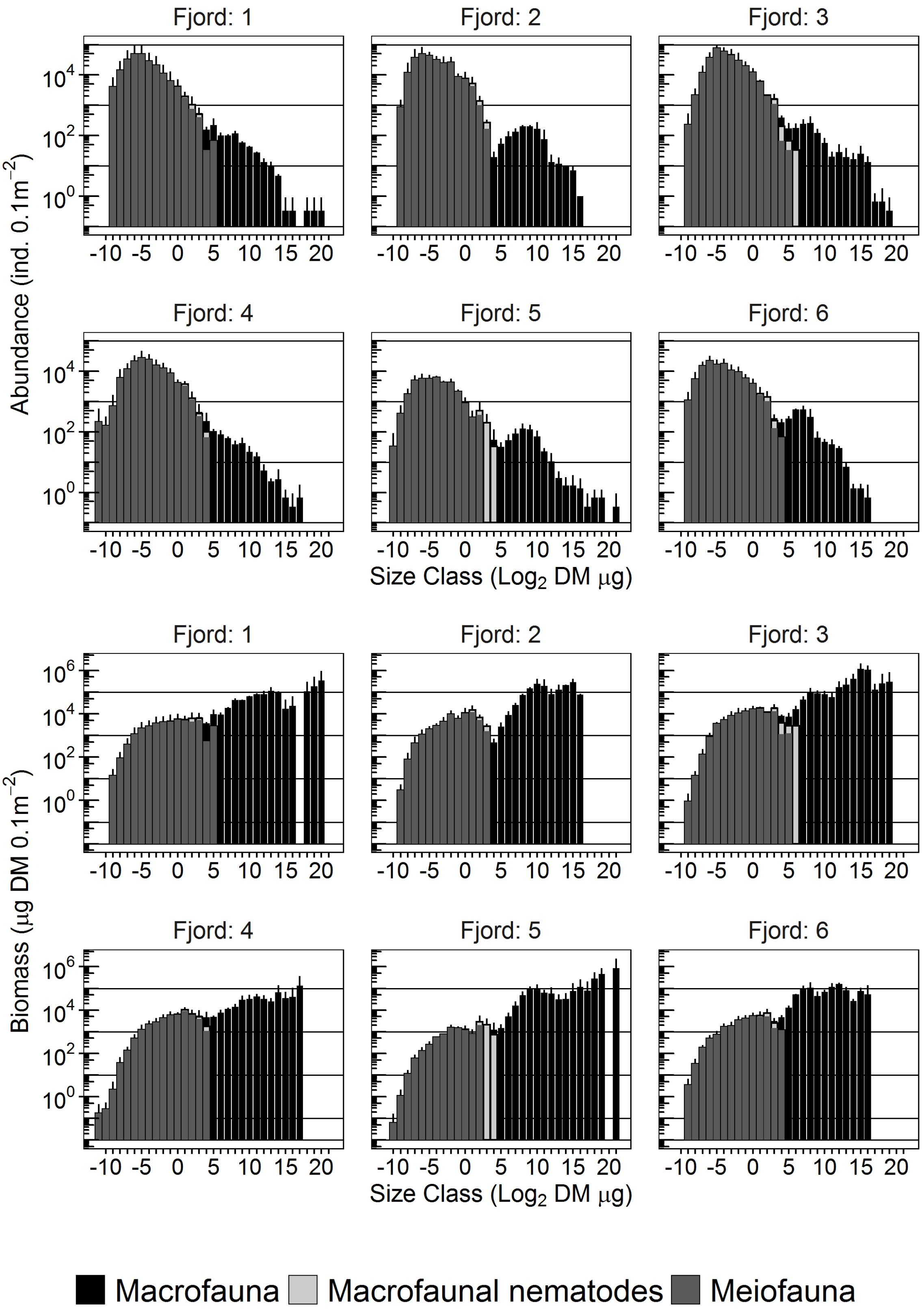

Figure S1 Abundance (top) and biomass (bottom) size spectra of the benthic community (meiofauna, macrofauna nemtatodes and macrofauna) in studied fjords . Mean for each group of organisms and mean+SD values of total biomass in each size class are presented.
